# Influenza in Asthmatics: For Better or for Worse?

**DOI:** 10.3389/fimmu.2018.01843

**Published:** 2018-08-10

**Authors:** Raja Veerapandian, John D. Snyder, Amali E. Samarasinghe

**Affiliations:** ^1^Department of Pediatrics, University of Tennessee Health Science Center, Memphis, TN, United States; ^2^Children’s Foundation Research Institute, University of Tennessee Health Science Center, Memphis, TN, United States; ^3^College of Graduate Health Sciences, University of Tennessee Health Science Center, Memphis, TN, United States

**Keywords:** allergic asthma, pandemic influenza, co-morbidity, eosinophils, mouse

## Abstract

Asthma and influenza are two pathologic conditions of the respiratory tract that affect millions worldwide. Influenza virus of the 2009 pandemic was highly transmissible and caused severe respiratory disease in young and middle-aged individuals. Asthma was discovered to be an underlying co-morbidity that led to hospitalizations during this influenza pandemic albeit with less severe outcomes. However, animal studies that investigated the relationship between allergic inflammation and pandemic (p)H1N1 infection, showed that while characteristics of allergic airways disease were exacerbated by this virus, governing immune responses that cause exacerbations may actually protect the host from severe outcomes associated with influenza. To better understand the relationship between asthma and severe influenza during the last pandemic, we conducted a systematic literature review of reports on hospitalized patients with asthma as a co-morbid condition during the pH1N1 season. Herein, we report that numerous other underlying conditions, such as cardiovascular, neurologic, and metabolic diseases may have been underplayed as major drivers of severe influenza during the 2009 pandemic. This review synopses, (1) asthma and influenza independently, (2) epidemiologic data surrounding asthma during the 2009 influenza pandemic, and (3) recent advances in our understanding of allergic host–pathogen interactions in the context of allergic airways disease and influenza in mouse models. Our goal is to showcase possible immunological benefits of allergic airways inflammation as countermeasures for influenza virus infections as a learning tool to discover novel pathways that can enhance our ability to hinder influenza virus replication and host pathology induced thereof.

## Introduction

Of the organ systems in the body that are exposed to the external environment, the pulmonary system is the most vulnerable due to its large surface area that accommodates a total of 10,000 L of air (containing an array of biologically active and inactive particles) daily. The upper and lower segments of the respiratory tract are bound by common structural and immunological components, allowing some infectious agents and secreted proteins in the upper respiratory tract to be transmitted to the lower respiratory tract. Therefore, the respiratory system must be considered as a whole when investigating disease pathogenesis and treatments, which is the basis of the “one-airway” concept.

While physical barriers and innate immune defenses serve to protect the lungs from infections and damage, susceptible individuals may develop responses to (often) innocuous agents over a period of time leading to the development of asthma, a major chronic disease of the airways (1, 2). Recent reports from the Centers of Disease Control and Prevention (CDC) estimate 1:11 children and 1:12 adults suffer from asthma (3). Sensitized airways dynamically react to inhaled agents as well as to respiratory pathogens, including viruses, that breach the barrier defenses.

Respiratory viruses, such as influenza A virus (IAV), respiratory syncytial virus (RSV), and rhinovirus (RV) are constantly circulating and can infect individuals of all ages, although the pediatric population is the most vulnerable (4). Simply based on high incidence of asthma, the probability of an asthmatic being infected with a respiratory virus is high, therefore, it is important to understand the host–pathogen interactions in an immunological background that is skewed toward type I hypersensitivity. A correlation between early-life infections with RSV (5, 6) and RV (7, 8) and the development of asthma has been demonstrated, albeit with caveats (9, 10). However, the interactions between IAV and asthma are less established, and therefore, the central focus of this review. This article is not meant to be a comprehensive review of either asthma immunology or that of influenza virus infections, but rather as a compilation of the state of the field surrounding co-pathogenesis of these immunologically distinct conditions that affect the respiratory system.

## Asthma

Symptoms of asthma were described in the Chinese literature as early as the twenty-sixth century BC (11), while the term “asthma” is derived from a Greek word used by Hippocrates which means “to exhale with open mouth, pant” (12, 13). Jean van Helmont’s description of the clinical symptoms in 1662 as “the lungs are contracted and drawn together” (14), was broadened in 1668 by Sir John Floyer who described asthma signs and symptoms, as well as prevention and prognosis and emphasized the importance of clean air (15).

In spite of improved understanding of disease pathogenesis, the incidence of asthma in the western world has increased over the past 40 years, and it is now the most common chronic disease in the world (16) affecting over 235 million people globally (17). Asthmatics can experience symptoms, such as wheezing, coughing, shortness of breath, and chest pain several times per day or week (18), often requiring preventative medical care. Ineffective treatment regimens have contributed to approximately 250,000 asthma-related deaths, more than 80% of which occur in low and lower-middle income countries (17). The yearly economic burden of asthma is estimated at $56 billion in the US alone (19).

**Figure 1:**
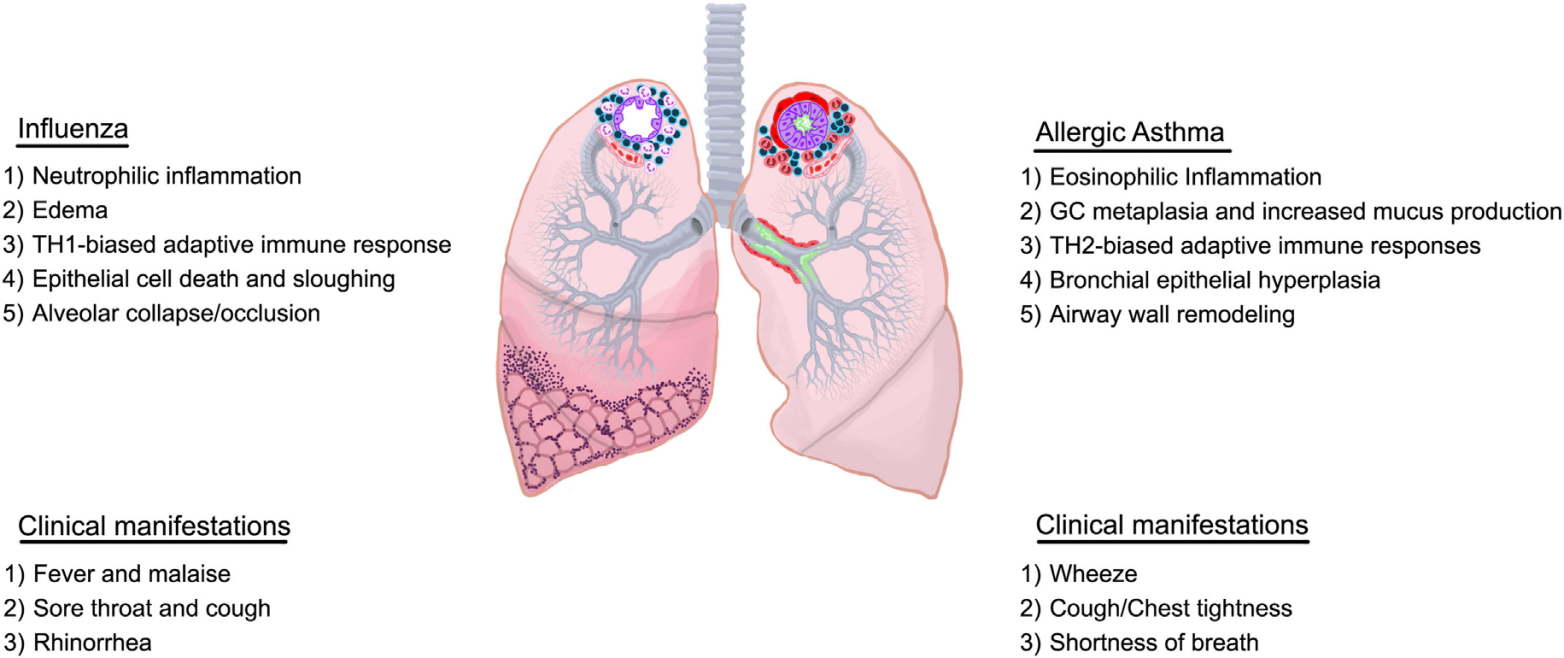
Overview of characteristics of influenza and allergic asthma. Immunological and structural components differ between influenza and allergic asthma although some overlap may exist in the clinical presentation.

Although the differences and similarities in disease etiology are unclear, various types of asthma, such as allergic asthma, exercise-induced asthma, cough variant asthma, occupational asthma, nocturnal asthma, and brittle asthma have been identified. Further classifications can be made based on the immune profile associated with the exacerbation (20, 21) adding to the complexity of this pulmonary condition. Some individuals do not fall under the definition of asthma issued by the WHO/NHLBI in 1995 to encompass the pathological and functional consequences of this condition (22). The most recent definition by Global Initiative for Asthma (GINA) is broader with more emphasis on symptoms rather than pathology (23) highlighting the shifted focus on heterogeneity of this complicated syndrome which has led to the stratification of patients by endotypes resulting in more personalized care. Allergic asthma is the most prevalent and results from exposures to extrinsic allergens (e.g., house dust mite and cockroach antigen, pollen, animal dander, and cigarette smoke). Airways sensitized to a particular allergen respond violently to subsequent exposures, resulting in asthma attacks, which can be fatal. Allergic asthma is characterized by eosinophilic airway inflammation (eosinophilia), airway hyperresponsiveness (AHR), and goblet cell metaplasia with associated increases in mucus production, epithelial shedding, and airway wall remodeling events (smooth muscle cell hyperplasia, subepithelial fibrosis, and angiogenesis) as depicted in Figure 1.

**Figure 2:**
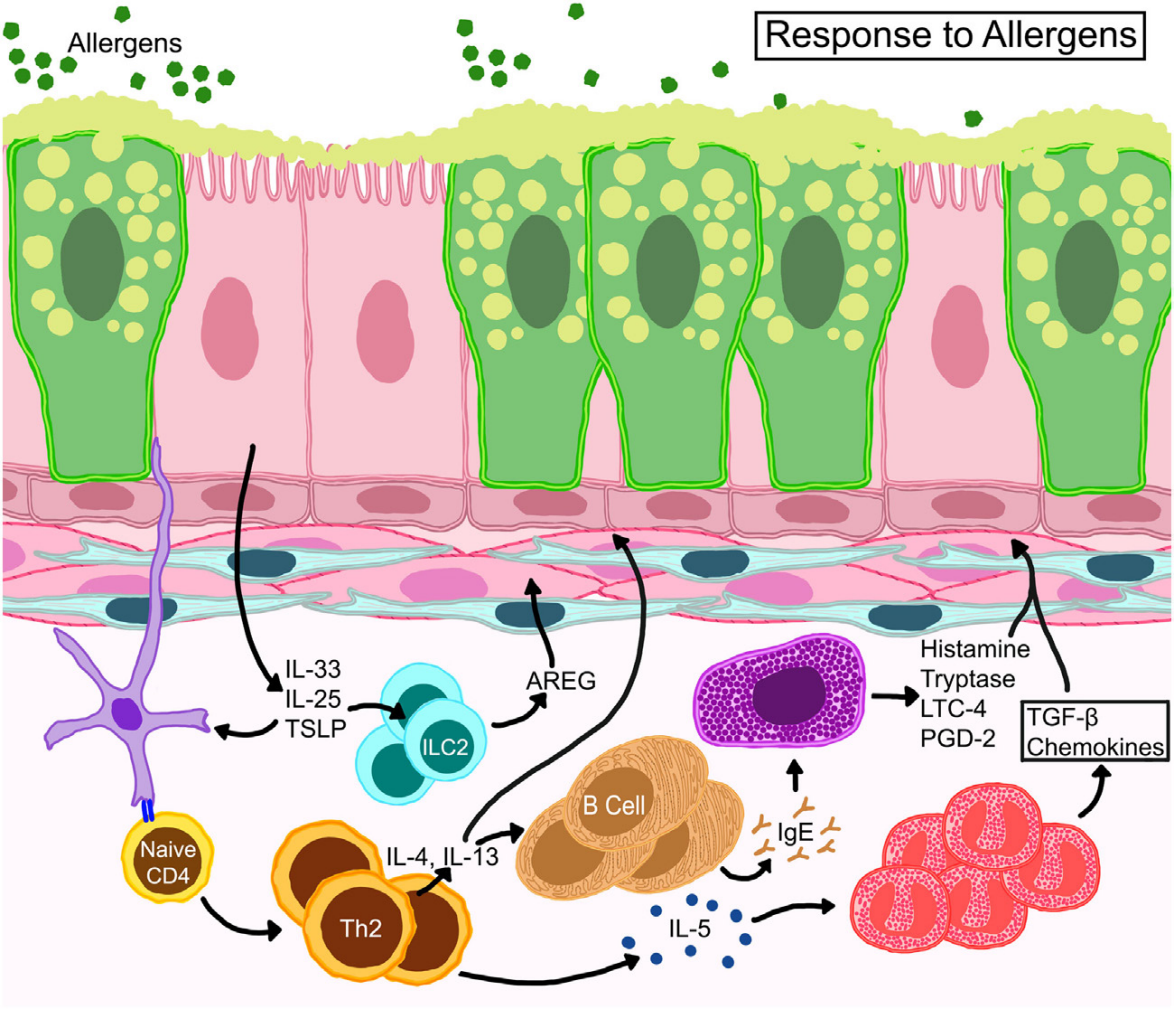
Schematic representation of basic immune responses in asthma. Epithelial cells release thymic stromal lymphopoietin, IL-25, and IL-33 activating allergen-activated dendritic cells to present antigen *via* MHC-II to T cell receptors of naïve T cells which convert to Th2 cells through expression of GATA-3 transcription factor. IL-4 and IL-13 from T_H_2 cells activate B cells for IgE synthesis. IL-13 also promotes goblet cell metaplasia and smooth muscle constriction. T_H_2 cells also control eosinophil development and survival through IL-5. Resident mast cells may become activated directly through allergen-specific IgE or indirectly through other myeloid cells and release cytokines, such as histamine, tryptase, leukotriene C4 (LTC4), and prostaglandin D2 (PGD2). Eosinophils release multiple growth factors and fibrogenic mediators that regulate architectural changes in the airways. Resident innate lymphoid cells (ILC) become activated to release amphiregulin (AREG) that may promote wound-healing or repair processes.

Allergic responses consist of an early phase which occurs immediately after allergen exposure, and a late phase which starts 6–9 h following allergen provocation. The early phase reaction is initiated through localization and signaling of high affinity Fcε receptors on mast cells (and others) bound to antigen-loaded immunoglobulin E (IgE). IgE-mediated degranulation of these cells causes the release of histamine, prostaglandins, leukotrienes, and reactive oxygen species, all of which can result in smooth muscle cell contraction, mucus hypersecretion, and vasodilation. Vasodilation and microvascular leakage cause plasma protein exudation into the airways leading to edema. Furthermore, plasma proteins bypass epithelial tight junctions and accumulate in the airway lumen interfering with mucociliary clearance (24). Plasma proteins, mucus, inflammatory cells, and shed epithelia form viscid plugs that compromise the luminal space and obstruct normal airflow (25). The late phase reaction, which includes AHR, involves the recruitment of various leukocytes. The contributions of each leukocyte to the pathogenesis of asthma have been characterized, but also a topic of ongoing investigations by various groups as reviewed in great detail elsewhere (26–30) and abridged in Figure 2.

The common symptomatologies of asthma (wheezing, chest tightness, cough, and breathlessness) can be generic and shared by other respiratory conditions, such as eosinophilic granulomatosis with polyangiitis (31), allergic bronchopulmonary aspergillosis (32), and chronic obstructive pulmonary disease (COPD) (33). In addition, the various phenotypes of asthma may have different clinical presentations. Wheezing is not unique to asthma as it can occur as a result of bronchial obstruction from a number of reasons (34), and children tend to wheeze more than adults (35). Wheezing is a common manifestation of viral infections in children (36–38), but not all children that wheeze develop asthma (35, 39). A reliable asthma diagnosis must be meticulously made (often by a pulmonologist) using measures of lung function and full history of characteristic symptom patterns confirmed by bronchodilator reversibility testing (23). Such rigorous testing required for a thorough diagnosis is often difficult to perform in children <5 years of age (40). Other confounders affecting the incidence and subsequent progression/alleviation include under- and over-diagnosis (41–43) and poor adherence to prescribed medication (40, 44).

### Respiratory Barrier Responses in Allergic Asthma

Asthma endotypes explain the various cellular cascades/pathways that manifest during different asthma phenotypes. The consensus between each endotype, however, is that the initiating trigger occurs at the bronchial epithelia (45, 46). The airway epithelium performs barrier and immune defense against foreign agents, such as allergens, viruses, or pollutants through active secretion of cytokines like thymic stromal lymphopoietin, GM-CSF, IL-1, IL-25, and IL-33 that attract and activate immune cells (47, 48). Dendritic cells (DCs) that intersperse the epithelium and capture inhaled allergens become activated by these cytokines and initiate adaptive immune responses triggering a cascade of events that involve numerous mediators and cell types including structural cells (49) (Figure 2).

It has long been observed that eosinophil counts in peripheral blood and bronchoalveolar lavage (BAL) fluid are higher in asthmatics compared to healthy controls (50). BAL fluid from patients with atopic asthma contain elevated levels of T_H_2 cytokines (51), including IL-5, which are strongly associated with eosinophilic inflammation (52). Eosinophils also serve as a source of numerous cytokines including IL-13 (53), that contribute to disease pathophysiology through increased AHR and mucus hypersecretion (54, 55). IL-13 is also produced by T_H_2 cells and type 2 innate lymphoid cells (ILC2s) that can produce amphiregulin (AREG) (56) a possible driver of wound healing responses in asthma. Lipid mediators, such as leukotrienes, contained in eosinophil (and mast cell) lipid bodies promote AHR and mucus hypersecretion (57) (Figure 2). While eosinophilia can correlate with asthma severity (58), not all patients with severe asthma have eosinophilia (59), highlighting the multifaceted and complex nature of this disease. In fact, heightened neutrophilia is a common finding in patients with severe/fatal asthma (60, 61).

## Influenza Virus

Influenza A, B, C, and D viruses are enveloped negative sense RNA viruses with segmented genomes that belong to the *Orthomyxoviridae* family. These viruses are morphologically spherical to ovoid and can range between 80 and 120 nm in diameter (62). While all four viruses can infect humans, IAV is the most common and pathogenic type. The outer envelope, acquired from the host, contains the prominent viral glycoproteins hemagglutinin (HA) and neuraminidase (NA) at a 4:1 ratio. Immunity against IAV is complicated because the virus undergoes antigenic drifts due to a non-proofreading polymerase, and antigenic shifts due to hosts that serve as mixing vessels for IAV with different sialic acid specificities. These features of IAV have resulted in four major pandemics and >50 million deaths worldwide as expertly reviewed recently (63).

Human infections result from influenza A and B viruses, with the former resulting in the majority of symptomatic infections. Viral transmission in humans largely depends on contact with viral particles contained in droplet nuclei released during sneezing or coughing. Droplet nuclei less than 5–10 µm containing infectious viral particles, can remain suspended in ambient air thereby allowing long-range transmission (64). Viral replication begins with viral entry which is dependent on HA interaction with sialic acids (sugars bound by glycosidic linkages to glycans) on the host cell surface. The low pH of the endosomal compartment triggers a conformational change in HA inducing membrane fusion and release of the viral genome into the cytoplasm followed by translocation of the viral RNA into the nucleus and hijacking of the host machinery to replicate its genome. Assembled virions bud off the host cell through actions of the viral NA activity. Functions of HA and NA in viral binding and budding are crucial for host adaptation and pathogenicity, while the antigenicity of HA is a criteria for novel strains with pandemic potential (65). Viral infection and replication cycle begins in the respiratory epithelium within 6 h, killing host cells within 12 h (66). Typically, in adult patients, viral replication peaks at 48–72 h post infection and clears after 1 week (66). Although influenza viruses circulate yearly, they effectively relegate host immunity by various immune evasion strategies as well as modifications to the genome.

Influenza includes both systemic (fever, malaise, and headache) and respiratory (cough, rhinorrhea, and breathlessness) symptoms. The minor overlap of symptomologies between asthma and influenza are likely due to architectural reasons as immune responses vastly differ (Figure 1). Immune responses against influenza viruses range from primary mucociliary barrier defenses to sophisticated adaptive immunity. Viral replication in the respiratory system can cause damage and induce death in epithelial cells which maintain the first line of defense against invading pathogens (67). Primary immune responses are initiated by epithelial cells and resident immune cells leading to the activation of adaptive immune responses that inhibit viral replication more effectively (68). While well-controlled immune responses are effective at viral clearance and regaining tissue homeostasis, continuous viral replication-induced tissue damage and ineffective inflammatory responses can lead to acute respiratory distress syndrome (ARDS), pneumonia, bacterial infections, and death (63, 69).

### Immune Responses at the Respiratory Barrier During Influenza Virus Infections

**Figure 3:**
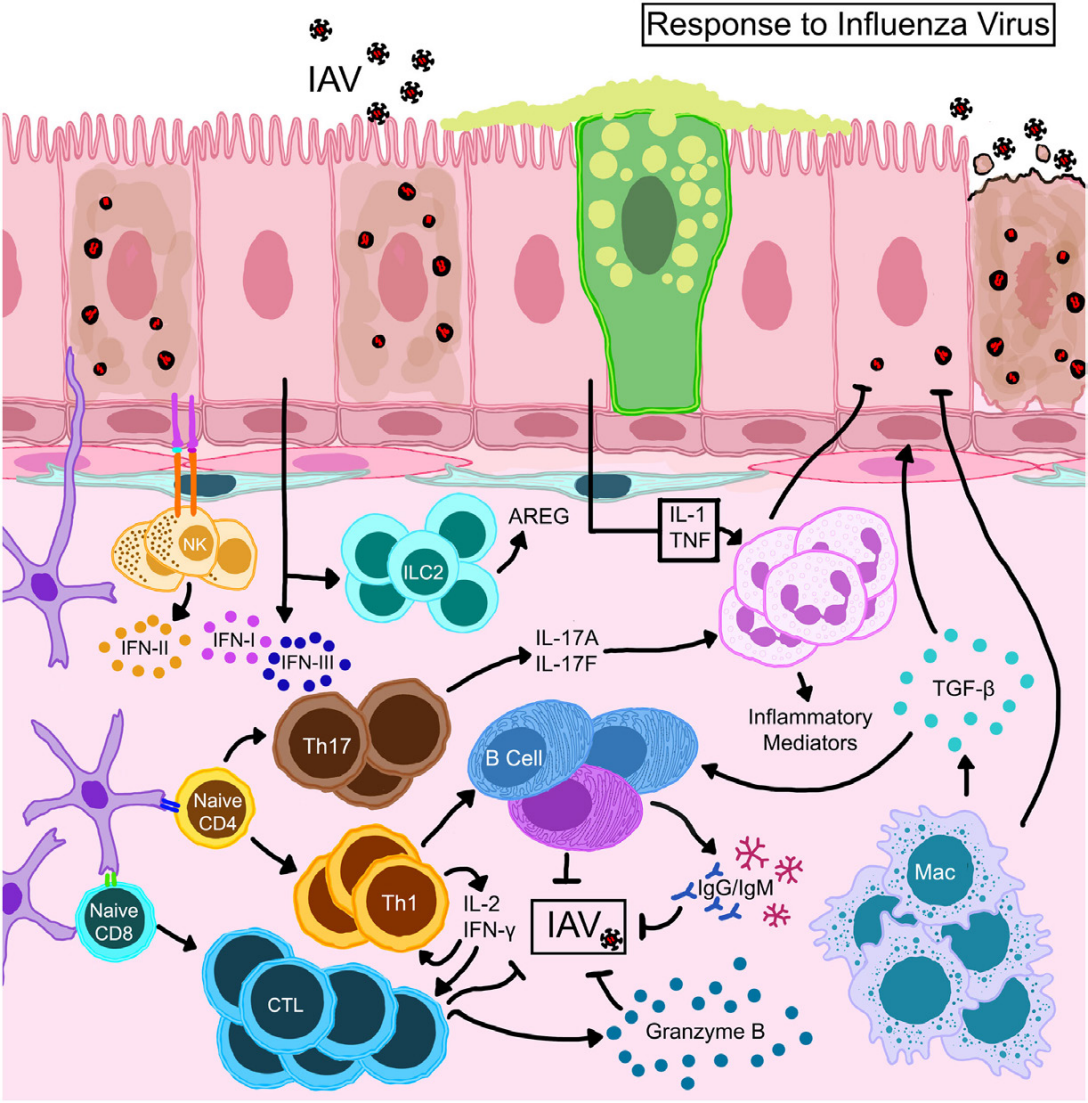
Schematic representation of immune mechanisms activated during influenza A virus (IAV) infection. IAV hemagglutinin binds to sialic acid residues on epithelial cells triggering immune responses. Natural killer cells that complex with antigens expressed on epithelial cells become activated to release type II interferon (IFN). Activated dendritic cells expressing CD11b^+^ CD103^+^ migrate to draining lymph nodes and prime T lymphocytes which differentiate into effector or memory cells. While CD8^+^ T cells directly kill virus-infected cells, CD4^+^ helper T cells direct the functions of resident/recruited cells through cytokine secretion. Transforming growth factor (TGF)-β produced by macrophages that are activated directly through TLR stimulation or indirectly by the local cytokine milieu and innate lymphoid cell (ILC)-driven amphiregulin (AREG) promote repair to the epithelial barrier. The damaged epithelial cells confer innate resistance by producing type I and type III interferons (IFNs) through stimulation of retinoic acid-inducible gene I.

Influenza viruses infect the airway epithelium and hijack the eukaryotic cellular machinery for rapid replication triggering both innate and adaptive immune responses (67, 70–72). Epithelial-derived inflammatory mediators as well as those produced by infiltrating leukocytes guide immune responses that ensue IAV infection (Figure 3). Functional responses of each cell type in the lung during IAV infection are interrelated by chemo/cytokine cues and antigen burden. Local interferons (IFN) are important to hinder viral replication; however, antiviral immunity can occur in the absence of IFN signaling (73). The release of cytokines traditionally associated with wound-repair [transforming growth factor-β (TGF-β)], homeostasis (IL-10), and allergy (IL-13) also occurs in response to IAV infection (68). While these cytokines can enhance anti-influenza immune responses and, therefore, be beneficial to the host during the tissue-repair phase of influenza, continuous availability in the lungs that can prolong viral pneumonia (74, 75) and increase susceptibility to bacterial infections (76) and asthma (77).

Although innate immune cells are important in antiviral immunity (78), their overzealous responses and high abundance can lead to host injury during influenza (79). Activated DCs present viral antigens to naïve/memory T cells initiating the adaptive immune cascade including antibody production by activated B cells (Figure 3). DC subsets have some diversity in their role as lymphocyte activators during influenza (68, 80). Macrophages reduce the viral burden by phagocytosis and efferocytosis of infected cells and also present antigen to boost adaptive immune responses. However, the reduction of macrophage numbers (81) and functions (82) in the lungs during influenza can occur as part of virus-induced inhibition of host defenses. Early neutrophil activation can reduce the antigen burden and improve adaptive immune responses (83). Cytotoxic T (84, 85) and natural killer (NK) (86) cells play a dominant role in controlling the infection and promoting viral clearance (Figure 3), after which inflammation resolves and tissue homeostasis can be regained.

## Influenza Pandemic of the Twenty-First Century

The first case of the 2009 “Swine Flu” (influenza) pandemic was identified in Mexico in mid-February, following which the CDC reported swine origin H1N1 influenza in two samples collected from patients in California (87). This virus replaced the circulating seasonal H1N1 virus and spread rapidly causing the WHO to declare this as the first influenza pandemic of the new millennium on June 11, 2009. The 2009 pandemic (p)H1N1 IAV is unique in that it arose in swine as a reassortant comprised of PB2 and PA genes from avian H3N2 virus, PB1 gene from human H3N2 virus, HA, NP, and NS genes from classical H1N1 swine virus, and NA and M genes from Eurasian H1N1 swine virus (88). Mutations changed binding efficacy and transmissibility of the strain, although pH1N1 was not highly pathogenic like H5 IAVs. Some mutants of pH1N1 containing amino acid changes in the HA gene (D222G, D222E, and D222N) were speculated to occur more frequently in severe cases of influenza (89), although resistance to oseltamivir (NA inhibitor) through H275Y mutation was not of significant health concern (90).

Unlike seasonal influenza strains to which infants and the elderly have the greatest susceptibility, pH1N1 virus caused more severe disease in school-aged children, adolescents, and adults ranging between 5 and 24 years of age (91). Although elderly individuals (>65 years of age) were less likely to become infected with this strain conceivably due to the presence of cross-reactive antibodies (92, 93), once infected, disease manifestation was severe in this age group possibly due to ineffective cellular immunity (91). Reported incubation period for pH1N1 was 1.4 days (94) when transmissibility was also greatest. Although viral infectivity decreased in adults between 3 and 5 days, symptoms associated with influenza (fever, malaise, headache, myalgia, cough, and rhinitis) presented between 5 and 10 days (95). Additional symptoms in children included otitis media, nausea, and vomiting (95). Although less severe than the past pandemics, the 2009 influenza pandemic caused more hospitalizations and respiratory complications than seasonal influenza (96). The WHO reported ~18,500 deaths from laboratory-confirmed influenza while recent estimates are as high as 203,000 deaths between March and December of 2009 (97). Mortality rates were lowest in children under 18 years (98) and the mean age at death was 37.4 years (99). Age groups that were at high risk for mortality differed from seasonal influenza, wherein, 51% of total deaths occurred in 20–49-year-old patients (100).

Nearly half of the hospitalized patients with Swine Flu had no reported co-morbidity (101), while various conditions were identified in the remaining patient population as a single underlying disease or as several that complicated influenza pathogenesis and increased the rate of hospitalizations; asthma was among these. Other conditions included pregnancy, obesity, COPD, diabetes, neurologic, and cardiac diseases (101, 102). Associated complications included viral and bacterial pneumonia, and ARDS often requiring mechanical ventilation (103, 104). Approximately a quarter of the hospitalized patients were admitted to the intensive care unit (ICU) during this pandemic, and the co-occurrence of at least one underlying disease increased the risk of ICU admittance (105). For example, neurologic conditions were most common in ICU-admitted children, while asthma was prominent in adults admitted to the ICU and obesity was a co-morbidity in both children and adults during this pandemic (105, 106).

## Asthma and the 2009 Swine FLU Pandemic

**Figure 4:**
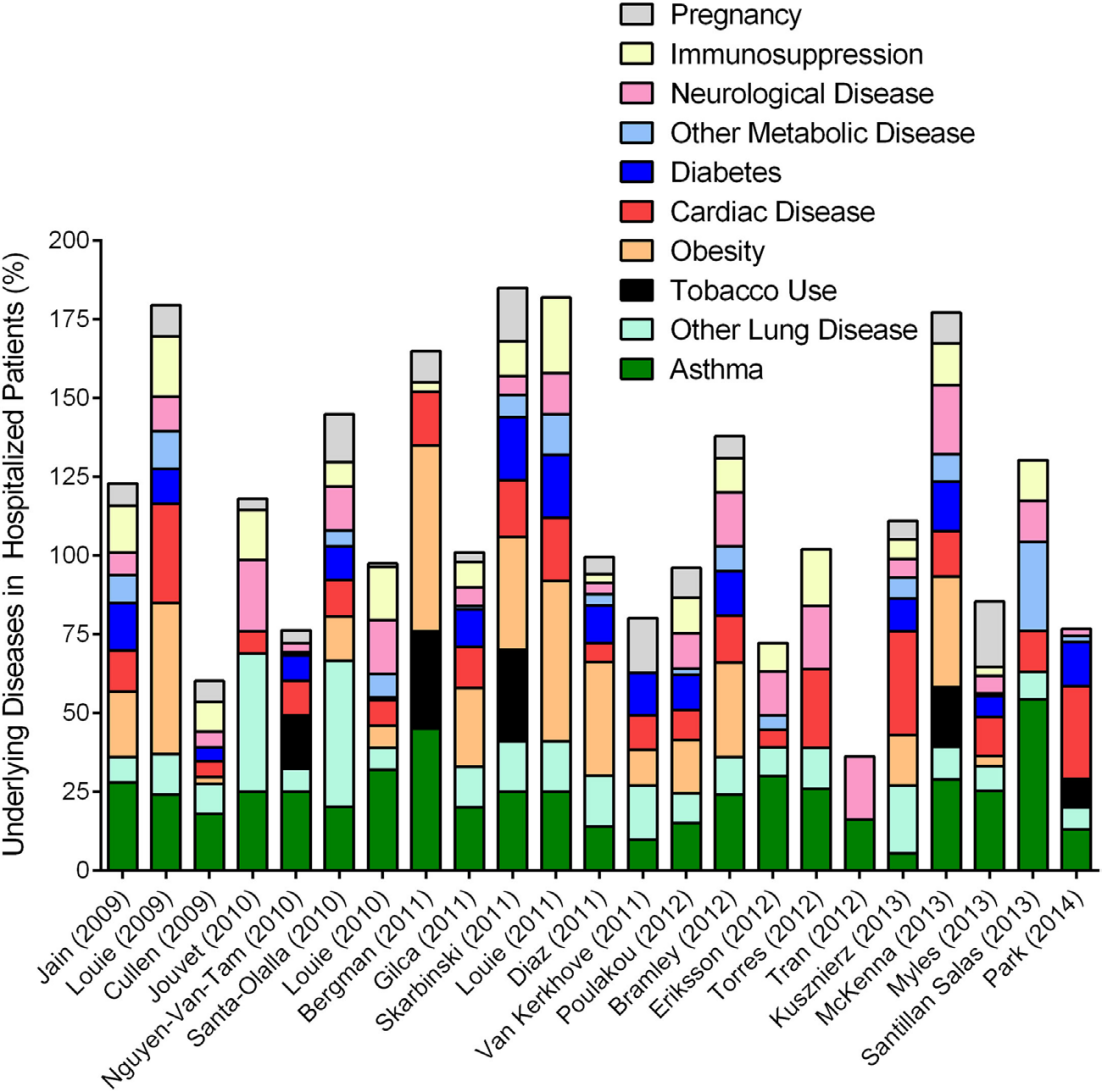
Overview of epidemiologic reports surrounding 2009 pandemic influenza in asthmatics. Epidemiologic findings reporting on the outcome of pandemic influenza in cohorts that included asthmatics were mined to calculate the percentages of patients with other reported diseases. Data from each manuscript were graphed to show the distribution of morbidities in patients hospitalized during the 2009 influenza pandemic. Values accounting for >100% indicate that patients within the cohort had multiple underlying disease conditions.

Unlike other influenza viruses that negatively impact “immunosuppressed” populations at either ends of the age spectrum, pH1N1 IAV strain caused hospitalizations in individuals between 18 and 50 years of age (104, 107). While the majority of these individuals were otherwise healthy (108), approximately 40% [and as many as 78% (98)] of the hospitalized patients had at least one underlying medical condition (Figure 4). Since other respiratory viruses (RSV and RV) clearly induce asthma exacerbations (109, 110), it came as no surprise when asthma was identified as a risk factor associated with hospitalization in both children and adults during the 2009 pandemic (104, 111, 112). Multiple investigations surrounding this pandemic reported on the incidence of co-morbidities that have been identified by the Advisory Committee on Immunization Practices (ACIP) to increase the risk of influenza morbidity. We reviewed a number of these clinical reports to obtain a better understanding of the frequency of common underlying diseases in hospitalized populations, and noted that asthma, obesity, and cardiac disease were among the most prevalent (Figure 4). While most other conditions have been noted previously, the identification that obesity increased the risk for severe outcome from influenza was novel (104, 113), and reproducible for this pandemic (Figure 4).

Asthma was an undisputed risk factor associated with hospitalization (Figure 4) affecting 10–20% of the hospitalized populations worldwide (102), and approximately a quarter of the hospitalized patients in the United States during the Swine Flu pandemic (114, 115). The increase in hospitalization among asthmatics may be attributed to a variety of factors including altered health seeking behavior (and in parents of asthmatics), heightened awareness due to media coverage, accelerated triage, and elevated physician precautions. Comparative analyses between seasonal and pandemic influenza concluded that the frequency of hospitalization and death as well as the profile of common underlying diseases, including asthma, were actually similar (116–119) although pH1N1 infections induced more severe disease in older age groups (120) and those with poorly controlled asthma (121) compared to seasonal strains. Therefore, greater attention to underlying diseases as risk factors during Swine Flu may have been due to the fact that pH1N1 induced more morbidity and mortality compared to the seasonal influenza virus strains that preceded it. Similarly, the 2017–2018 influenza season drew more attention based on the high mortality associated with this H3N2 virus strain compared to previous seasonal strains (122).

Lung diseases are often complex and multifactorial, and, can sometimes overlap. COPD was identified as a risk factor associated with worse outcomes (123). Asthma and COPD may share some phenotypic overlap in adults, but remain immunologically and physiologically distinct conditions. Therefore, influenza outcomes reported in combination for asthma and COPD may not necessarily reflect actual circumstances of either disease, unless categorized as asthma-COPD overlap syndrome (122). In one report, the physician diagnosis for asthma and ICD-9 code for acute asthma did not correlate (120), and the criteria for the identification of “asthma” in patients were not specified in most reports that focused on asthma incidence in their respective cohorts. Specific diagnosis for asthma requires common allergen testing, serum IgE testing, successfully executed spirometry at baseline and often after exercise, symptom reversal after treatment with short acting beta agonists (40, 45) etc., that require a specialist. As such, data that relied on self-reporting or clinician’s notes at triage must be carefully interpreted (124–126) as wheezing non-asthmatics may have been categorized incorrectly as asthmatics. Although environmental pollutants are known to complicate asthma (127) and influenza (128), information on tobacco smoke exposure was only provided in a small number of these reports (Figure 4). In addition, as noted in our compilation of the previous literature, most patients had more than one underlying condition (Figure 4), although this was only considered by some authors (98, 111, 114, 129, 130). Complex interactions between asthma and other underlying conditions, including obesity (131), diabetes (132), and cardiac disease (133) for example, are only beginning to be elucidated. However, these disease–disease interactions are likely to impact host responses to IAV infection and thereby affect the patient’s medical outcome. Similarly, since IAV infections can induce exacerbations of the underlying disease (cardiac, diabetes, etc.), it may be likely that the number of severe cases due to influenza were actually underestimated as patients visiting medical practices may not have been tested for influenza when the primary complaints were non-respiratory.

The age of onset and endotype often determines the chronicity of asthma and maintenance medication use. Patients that were not on long-term therapeutics to control asthma symptoms had a higher incidence of pH1N1 infection (120, 121, 134, 135). While the pH1N1 virus did increase the number of asthma exacerbations compared to seasonal strains (126), virus infection did not correlate to the severity of asthma symptoms (126). Higher rates of infection and hospitalization of children also corresponded with higher rates of admission to pediatric intensive care units (PICU) during the 2009 influenza pandemic. Lung disease (43.9%) and asthma (25%) were among the most common co-morbidities in these critically ill children (116). Torres et al. noted that asthma was among the risk factors associated with PICU admission and mortality in children under 24 months during the Swine Flu pandemic, as was co-infection with RSV (125). However, since it is difficult to diagnose asthma in children at this age (40, 45) as noted by other investigators (136), and RSV and IAV can induce wheezing in young children, the actual lung disease in these critically ill, very young, pediatric patients may not have been asthma.

Asthmatics are generally considered inept at countering virus infections effectively due to immune bias. Information largely focusing on RSV and RV have shown that asthmatics have reduced type I IFN responses during respiratory infection (137). Whether asthmatics were more likely to be infected by pH1N1 than non-asthmatics, or whether there were differences in the viral replication or clearance once infected, were not clear during the pandemic, largely because most data were from the hospitalized populations without matched controls and because these questions are not easily addressed in studies not designed to do so. Kloepfer et al. noted that children with asthma were more likely to become infected with pH1N1 virus (134). While this conclusion was drawn from the identification of pH1N1 in 10% of the 346 nasal swabs collected from 193 children (with and without asthma), it should be noted that 62 and 12% of these samples tested positive for RV and enterovirus, respectively, and an additional 13% were positive for pH1N1 and RV (134). Similarly, RSV coinfections were found in 43% of children infected with pH1N1 (138). Since these respiratory viruses are known to exacerbate asthma, and asthma attacks were a salient reason for hospitalization (121, 129), coinfections could have escalated the incidence of hospitalization of asthmatics during this pandemic. Although very few reports provided information on the rate of viral coinfections, seasonal overlap between RV, IAV, and RSV may promote virus–virus interactions thereby altering host–viral interactions that govern infection. Additionally, since pH1N1 infectivity and replication in primary bronchial epithelial cells from adult asthmatics were similar to that in cells from healthy donors (139), it is possible that an age-related difference in pH1N1 may occur at the cellular level.

Disease severity during the Swine Flu pandemic was further marked by ICU admittance and death (140). Intriguingly, however, a number of reports suggested that asthmatics had less severe influenza morbidity compared to non-asthmatics (102, 105, 113, 115, 119, 129, 130, 136, 141, 142) with decreased risk of requiring ICU admittance, mechanical ventilation, and death. Analysis of data from 1,520 pH1N1 confirmed patients in the UK also showed that asthmatics had a reduced risk for severe outcome from influenza (143). Asthmatics were also more responsive to early antiviral (136) and corticosteroid (144) intervention. Although steroid use has been proposed as a reason for favorable outcome in asthmatics during the 2009 influenza pandemic (130), corticosteroid treatment has also been associated with increased risk of mortality, nosocomial infections, and prolonged mechanical ventilation and ICU length of stay (145, 146). Asthmatics that are on steroid therapy may have been at increased risk for pH1N1 infection due to their transient immunosuppressed state. Therefore, the impact of steroid treatment during the 2009 influenza pandemic is ambiguous and may have enabled the likelihood of infection/complications or protection against severe influenza. Since patients with asthma and COPD were found among those admitted to the ICU (111, 114, 116, 117, 136, 147), and among those that died (98, 100), it is clear that the interaction between asthma and pH1N1 was complex, and may have been altered by asthma phenotype and endotype as suggested by animal studies described below.

Vaccination and antivirals are available protectants prior to and during IAV infections. Annual vaccination against influenza infection is recommended by the WHO for all individuals but especially for pregnant women, children, and the elderly, and those with underlying health conditions. In spite of these recommendations, less than ~50% of the US population seeks the annual influenza vaccine (148). While vaccine efficacy may vary based on viral evolution and individual immune status, vaccines are beneficial as they are effective for symptom resolution and may help to slow down the rate of disease progression through at least a polyclonal non-specific humoral response during infections, and as such, better sought than not. Antiviral medications are available to mitigate the impact of IAV, however, investigations into the use and effectiveness of these medications during Swine Flu are limited (149). In general, antivirals (primarily oseltamivir) were provided to >80% hospitalized patients and their use were similar between asthmatics and non-asthmatics during the last influenza pandemic (105, 113, 114, 125). As such, the observation that asthmatics were less likely to develop complications from pH1N1 infections (102, 105, 113, 115, 142) was unlikely due to changes in viral load. In support of this, Oshansky et al. found no relationship between viral load and hospitalization in a pediatric patient population which included asthmatics (150).

The use of clinical data factors as both a strength and weakness in exploring outcomes of infection. Variability in available and reported data can contribute to differences in conclusions as evident in the literature that focused on asthma in hospitalized patients during the 2009 influenza pandemic. Since the combined percentages of diseases in some cohorts were over 100% (Figure 4), it should be noted that most hospitalized patients had more than one underlying health condition. Identification of high-risk patients and understanding the pathophysiology of disease and complications thereof during influenza are important for pandemic preparedness. However, misclassification, administration of controller medication to patients that may not have severe (eosinophilic) asthma, viral-induced exacerbations, coinfections, other underlying conditions, and environmental toxins may adversely affect the outcome of influenza in asthmatics. Most often, however, information on the phenotype and endotype of asthma, history of controller medications, time since last exacerbation, BMI, prominent granulocyte during disease (eosinophilic/neutrophilic/mixed/paucigranulocytic), tobacco smoke exposure, quality of air (pollutants, allergens, etc.), and influenza vaccination history, are not available in the clinical reports, and difficult to obtain for any epidemiologic report due to the nature of medical records, but would be ideal to provide a complete landscape of how asthmatics respond to circulating influenza viruses. As such, animal model systems are invaluable to delineate the mechanisms of host–pathogen interactions to understand disease interactions and outcomes in patients during the convergence of these two immunologically distinct diseases.

## Proposed Mechanistic Insights on the Pathogenesis of Asthma and Influenza from Animal Models

Lower respiratory tract infection by 2009 pH1N1 virus has been associated with asthma exacerbations as well as viral pneumonia (135, 151). Patients who developed acute pneumonia from pH1N1 infection had markedly greater levels of T_H_2 cytokines (IL-4, IL-5, and IL-13) in the serum compared to those that did not (152). The increase in these canonical T_H_2 cytokines may suggest the initiation of the wound healing process as mediators associated with T_H_2 responses are transiently elevated in the lungs at the conclusion of the H1N1 infection cycle in mice (139, 153). Complications with acute pneumonia also produced bronchial mucus plugs with elevated levels of eosinophils and neutrophils, even in patients without allergy (154). Bronchial epithelia are susceptible to IAV infection and can become apoptotic with increased viral burden. Interestingly, primary bronchial epithelial cells from asthmatic donors were resistant to the cytopathology induced by pH1N1 while those from healthy donors were not (139). As such, the interactions that may occur between influenza virus and the allergic host may depend on a variety of factors, including the endotype of asthma, the immune status, other underlying diseases, and the architectural state of the airways at the time of virus infection. Furthermore, healthy airways, allergen sensitized airways, and airways that were recently exposed to allergens are three independently unique landscapes that would differ in response to IAV infection. Most animal studies of asthma and influenza have focused on the impact of IAV as a trigger for subsequent allergic responses (77, 155–157). However, asthma and influenza are complicated diseases that involve numerous immune and structural cells of the respiratory tract as depicted in Figures 2 and 3. Furthermore, since asthma is multifaceted and dynamic with structural changes that can occur between exacerbations, the way in which an asthmatic would react to viral infections cannot be effectively predicted without animal models that can recapitulate the clinical findings both showing asthma exacerbations induced by IAV infection as well as the protection from virus-induced severe complications as discussed above.

**Figure 5:**
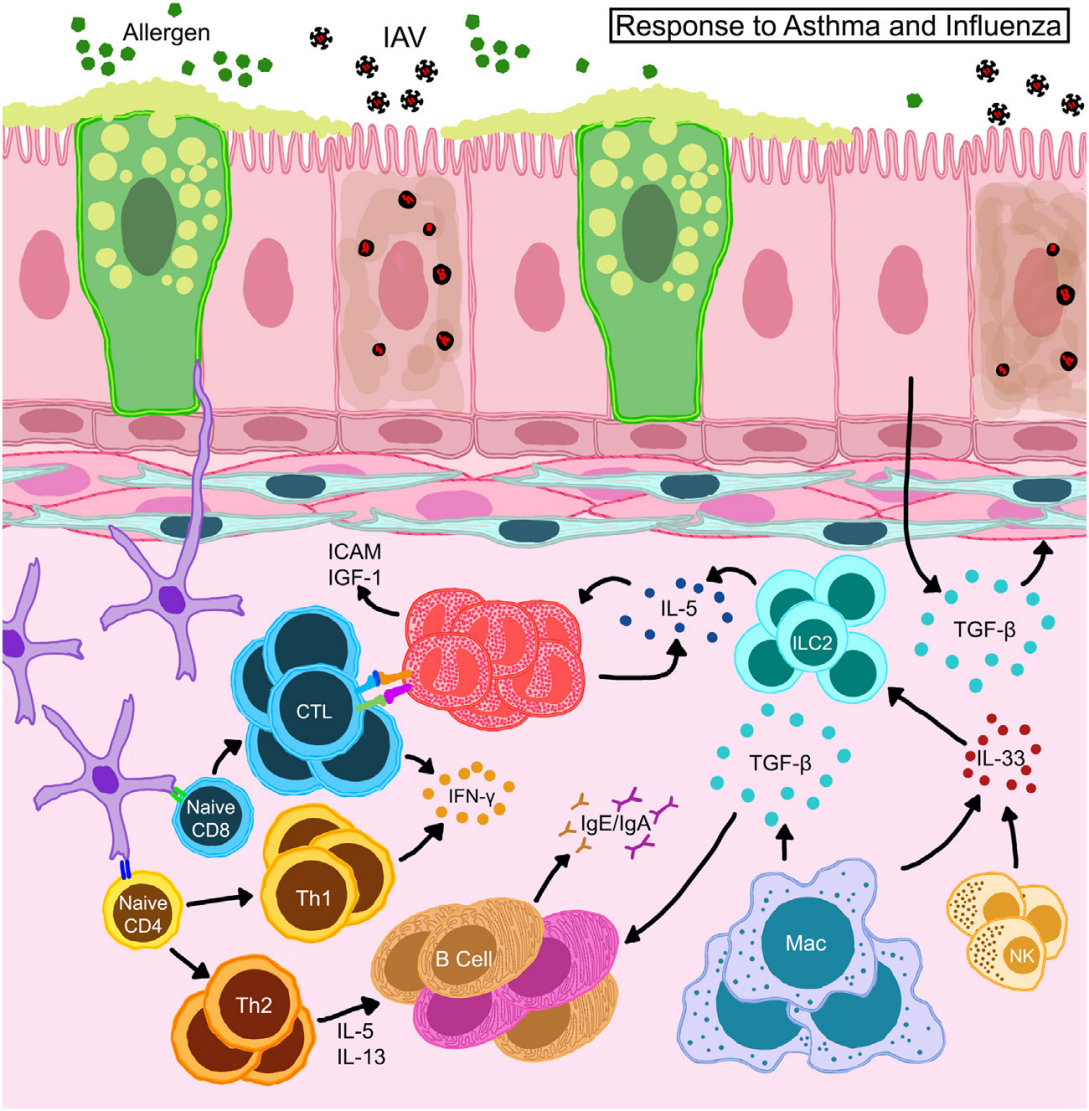
Schematic representation of immune responses in allergic hosts during influenza A virus infection. Bronchial epithelia in allergic hosts upregulate intracellular adhesion molecule (ICAM-1) and insulin-like growth factor (IGF-1) to recruit/activate immune mediators. Alveolar macrophages and natural killer (NK) cells release large amounts of IL-33 and trigger innate lymphoid cells (ILCs) to release IL-5 thereby induce eosinophil accumulation and survival *in situ*. Eosinophils engage in the activation of CD8^+^ T cells in the draining lymph nodes as well as on site to help enhance antiviral cellular immunity in the allergic host. Locally derived transforming growth factor (TGF)-β inhibits virus-induced pathology and may promote *in situ* IgA production. Interferons (IFN) released by T cells are important to heighten the “antiviral” state in the microenvironment to safeguard structural and immune cells from virus-induced cytotoxicity.

The number of mouse models that use mice with established allergic inflammation for subsequent infection with IAV are limited (139, 158–161), but have already provided important information regarding the pathogenesis of IAV in asthma relevant to the Swine Flu pandemic (Figure 5). Ishikawa et al. found that ovalbumin-induced asthma protected mice from influenza, a phenomenon that they attributed to NK cells (161) (Figure 5). Using a clinically relevant mouse model of severe asthma with fungal sensitization, we showed that acute allergic inflammation induced by allergen provocation protected mice from pH1N1-induced influenza, while chronic remodeling that resulted from fungal challenge made mice susceptible to influenza morbidity and host pathology (139) highlighting the impact of the temporal association between allergen provocation and viral infection in disease outcome during asthma and influenza. The discovery that mice with heightened allergic responses in the lungs had less severe influenza were later confirmed by other groups (158, 160, 162) all using different allergens suggesting that this outcome is common to allergic asthma. Ovalbumin-induced allergic airways disease was resistant to lethal pH1N1 infections (158) through mechanisms that involved the TGF-β pathway (163) (Figure 5). As a mediator of remodeling, TGF-β is involved in tissue repair and re-modeling of the respiratory tract through stimulation of matrix protein production, epithelial proliferation and differentiation (164). It is also an immunoregulator that promotes differentiation of T_regs_ that suppress (165) or enhance (166) adaptive immunity to influenza viruses. These model systems now provide an avenue to explore the epidemiologic findings surrounding asthma during the Swine Flu pandemic. However, further advances are necessary to investigate the function of genetic factors influencing asthma and subsequent viral infections to investigate the gene:environment impact on co-morbidity that could have influenced some asthmatics during the Swine Flu pandemic.

Animal models described above have been able to contribute mechanistic insights into the complex interactions between allergic inflammation and IAV infections. Viral clearance is a crucial step in initiating recovery from influenza. The role of CD8^+^ T cells as mediators of viral clearance through targeted removal of infected epithelia is well established (85). Our characterization of antiviral responses in hosts with acute and chronic asthma showed that viral clearance was enhanced in mice with acute allergic asthma which also had elevated influenza-specific CD8^+^ T cells (139), which we subsequently determined to be due to putative antigen-presenting functions in eosinophils that enhanced cellular immunity (167) (Figure 5). Others have shown that CD11b^+^ DCs are better at CD8^+^ T cell activation in context of asthma and influenza co-morbidity (160) (Figure 5). Neutrophils provide additional support for cellular immune responses during influenza by guiding the migration of CD8^+^ T cells into the lungs (168). While the function of neutrophils in asthma and influenza co-morbidity has not been investigated to date, it is an area of interest especially in consideration of severe asthmatics with neutrophilia. Similarly, since the role of CD8^+^ T cells in the pathogenesis of asthma is not fully elucidated (169), the development and characterization of CD8^+^ T cell responses in the context of asthma and viral infections in humans would be of great interest.

The rapid antigenic changes in circulating influenza viruses reduce natural immunity against these viruses and hinder the development of effective vaccinations. Although the influenza vaccine is highly recommended for patients with asthma per ACIP and the WHO, compliance low. While total B cell numbers in the pulmonary mucosa are similar between allergic and non-allergic mice after pH1N1 infection (158), allergy-specific antibodies dominate over anti-influenza antibodies in the serum and mucosa of allergen-exposed pH1N1-infected mice (170) suggesting that pre-existing allergy does not readily promote antiviral humoral immune responses (Figure 5). B cell populations in lymphoid organs were not markedly different between virus-infected mice with/without allergic airways disease, however, antibody producing B cells in the lungs were elevated in co-morbid mice (170) indicating the importance of *in situ* mucosal immune responses in pulmonary co-morbidity. Therefore, examining the development and function of B cell responses during asthma and influenza co-morbidity may help to delineate effective vaccination strategies for patients with underlying chronic lung diseases.

The intricate acute and chronic changes in airways that occur over time make asthma a difficult syndrome to model in mice. As such, most mouse models of asthma rely on exposures (often initiated through adjuvants) localized to the lungs to initiate acute features of asthma largely relying on granulocytic inflammation as a hallmark. The above referenced mouse models of asthma and influenza also predominantly focused on IAV infections in mice with “acute asthma” primarily because they were built to reproduce the clinical findings that asthmatics with exacerbations were protected from severe influenza disease. Very few mouse models are able to produce airway wall remodeling as a chronic feature of asthma (171). We have also shown the opposite clinical finding that some asthmatics (including COPD patients) did suffer from severe influenza requiring ICU admittance using a mouse model of chronic asthma and influenza (139) underscoring the importance of attention to temporal associations. Additional mouse models and mechanistic evidence exploring chronic asthma and influenza are necessary in order to fully elucidate the full spectrum of disease–disease interactions.

The strong gene:environment association that occurs in asthma also contributes to disease pathogenesis and response to viruses. For example, a single nucleotide polymorphism in *ORMDL3* has been linked to increase susceptibility to RV-induced wheezing and subsequent asthma diagnosis (172). However, a model of RV-infection using a transgenic mouse showed that ORMDL3 overexpression improved antiviral defense thereby limiting viral replication and reducing inflammation (173), suggesting that the gene:environment interactions may be abstruse even for genes identified by genome-wide association studies. Recognition of similar genetic involvement in the pathogenesis of influenza in allergic hosts may be important to delineate why some patients fair better/worse during influenza virus infections. The common perception that asthmatics are unable to launch effective T_H_1 responses against respiratory viruses stems largely from investigations surrounding RV (174). A mixed cytokine profile has been demonstrated in allergic mice that are infected with pH1N1 wherein these animals had T_H_1, T_H_2, and T_H_9 responses (158). Immune responses to a respiratory virus are dictated by a sophisticated amalgamation of genetic, immune memory (past experience), and topography of the pulmonary milieu, and unlikely to be of comparable strength and phenotype between viruses. While the 2009 pH1N1 infections resulted in asthma exacerbations (similar to other viruses), the less severe influenza disease outcomes suggest that the cytokine storm induced was protective against viral pathologies further emphasizing that antiviral immune responses and consequences thereof are unique and situation-dependent.

## Conclusion

Asthma and influenza are common conditions that affect millions worldwide. Although pathogens like RSV and RV are known to initiate and exacerbate asthma, the relationship between asthma and influenza was ambiguous. The recognition that asthma was a risk factor associated with hospitalization during the 2009 influenza pandemic put these two diseases in the spotlight as a duo for poor outcomes. The notion that hypersensitivity-based immune responses may be protective to the host during pathogen encounters was first suggested by Arthur Varner (175), and confirmed during the Swine Flu pandemic wherein asthmatics were less likely to suffer from complications related to severe influenza. Mechanisms that may have resulted in this unexpected outcome were identified using mouse model systems wherein the immune profile associated with acute allergic exacerbation was found to have enhanced antiviral properties. In this review, we focused on the complexities of these immunologically distinct diseases both independently and together to highlight the intricacies associated with understanding health conditions that presently affect the population. As new discoveries are made that emphasize different endotypes of asthma, it is important to investigate how these immune responses impact invading pathogens as these contextual investigations will benefit our goal of improving personalized medicine.

## Author Contributions

RV and AS wrote the manuscript. RV and AS analyzed literature and produced Figure 4. JS prepared the schematics. All authors read and approved the final version.

## Conflict of Interest Statement

The authors declare that the research was conducted in the absence of any commercial or financial relationships that could be construed as a potential conflict of interest.
